# The association between meal-based diet quality index-international (DQI-I) with obesity in adults

**DOI:** 10.1186/s40795-022-00654-0

**Published:** 2022-12-27

**Authors:** Esmail Alipour Nosrani, Maryam Majd, Elham Bazshahi, Fatemeh Mohtashaminia, Hanieh Moosavi, Reza Ramezani, Hossein Shahinfar, Farhang Djafari, Sakineh Shab-Bidar, Abolghassem Djazayery

**Affiliations:** 1 Department of Nutrition, Science and Research Branch, Islamic Azad University, Tehran, Iran; 2 Department of Community Nutrition, School of Nutritional Sciences and Dietetics, Tehran University of Medical Sciences (TUMS), Tehran, Iran; 3 Department of Nutrition, School of Public Health, Iran University of Medical Sciences, Tehran, Iran

**Keywords:** Meal-based, DQI-I, Obesity

## Abstract

**Background and objective:**

Due to the growing global trend of obesity, it is necessary to study the diet quality as a modifiable factor to reduce the dangerous consequences of obesity. Therefore, the aim of this study was to evaluate the association between meal-based diet quality index-international (DQI-I) with obesity in adults.

**Methods:**

This cross-sectional study was performed on 850 men and women in Tehran (aged 20–59 y). Dietary intakes were assessed using three 24-h dietary recalls. Meal-based Diet quality was assessed based on the construction of DQI-I. The total DQI-I score ranged from 0 to 100, with higher scores denoting better diet quality. Multiple linear regression analysis was used to examine the association of DQI-I and BMI in each meal and Logistic regression analysis was used to examine the association of DQI-I and obesity in each meal.

**Results:**

The mean (± SD) of age, body mass index (BMI), waist circumference (WC) and waist to hip ratio (WHR) were 42.35(± 10.90) years, 27.32(± 5.61) kg/m2, 89.09 (± 12.04) cm and 0.86 (± 0.11), respectively. In none of the meals, after adjusting for confounders, no significant difference in BMI was observed in the both women and men groups. After controlling of confounders, there was not any relationship between meal-based DQI-I and BMI resulted from multiple linear regression analysis also there was not any significant association between meal-based DQI-I and obesity resulted from Logistic regression analysis.

**Conclusion:**

In this study, we did not find any significant association between meal-specified DQI with obesity. To reach the better evaluation, more prospective studies with large sample size are needed.

## Introduction

The global prevalence of obesity is increasing around the world and further increases are estimated in the future [1]. The number of obese people has doubled between 1980 and 2015 [2]and in Iran, 25.8% of adults were obese in 2016 [3]. Adult obesity is associated with many health consequences including diabetes, cancer, respiratory and cardiovascular diseases [2, 4].

Diet, sedentary lifestyle, genetic, environmental, neurological, physiological, biochemical, socio-cultural and psychological factors are elements that contributing to obesity [5]. Diet as one of the modifiable factors plays a crucial role in a people's health and prevention of non-communicable diseases [6]. There are numerous evidences that suggest an association between different components of diet including energy, food groups and nutrients with obesity [7–11]. Because the interaction between foods and nutrients is important, the assessment of overall dietary patterns is more useful than single nutrients in relation to obesity [12].

Dietary Quality Indices (DQIs) are designed to examine the overall diet and categorize individuals based on the extent to which their eating behavior is healthy [13]. The diet quality index-international (DQI-I) is one of these indices that emphasizes on four major aspects including high-quality, healthy diet, i.e., variety, adequacy, moderation and overall balance [14]. Kim et al., found that the DQI-I is a useful tool to assess the dietary quality [14]. In a study in Iran, Ebrahimi et al., also indicated that DQI-I may be more applicable than HEI for evaluating Iranian nutrient adequacy [15].

Few studies have done in relation to the association between the diet quality index-international with obesity and different results have been obtained, for example In Iran, Asghari et al. did not find a significant relationship between DQI and obesity [16, 17], but this association has not yet been assessed at the meal level.

The association of dietary patterns and diseases has been considered well so far, but little attention has been paid to meals [18]. Foods are consumed as meals and snacks, and thus investigation of diet quality across meals is a practical approach to help explain important diet–disease relationships [19]. Recent studies have suggested that meal-specific dietary properties such as meal timing and frequency and composition of the diet at each meal may be associated with multiple health outcomes [20]. It was reported that there is an association between meal patterns and weight status [21, 22]. For example Breakfast as the first meal of the day contributes to an overall healthy dietary pattern, better nutrient intake, and diet quality [23]. Recognizing meal-based diet quality indexes may help the public to achieve the recommended daily intake of foods and nutrients and adhere to dietary guidelines. We aimed to investigate the association between meal-based diet quality index-international (DQI-I) with obesity in apparently healthy adults referring to the health centers in a sample of Iranian adults in Tehran, as a representative population of Iran.

## Subjects and methods

### Study design

A total of 850 healthy adult men and women, aged 18 to 59, who were willing to participate in this cross-sectional study, were recruited from health care centers of Tehran, via a two-stage cluster sampling using advertisement, spreading of flyers in common places and information sessions at health care centers about the goal and the benefit of the examination. First, the city was split into five regions north, east, south, west, and center. A list of all existing health care centers was provided and then eight health centers were randomly chosen from each region for a tally of forty health centers. Ultimately, the sample size (*n* = 850) was divided by 40 to get the number of subjects in each health center. To be noted that we recruited people from the health centers of Tehran affiliated to the Health Bureau of the Municipality of Tehran. Indeed, Tehran is the Capital of Iran and has a multiethnic population, and in health research in Iran, population of Tehran are considered as a representative of Iran.

Based on the prevalence of obesity and overweight in the adults of Tehran (65%), an error coefficient of d=0.04 and at α level of 0.05, a sample size of 546 people was calculated $\left(n=\frac{(z^2-p(1-p)}{d^2}=\frac{(1.96{)^2\;}\ast\;0.65\;\ast\;0.35}{\left(0.04^2\right)}\right)$. Due to the potential exclusion of participants, the sample size was multiplied by 1.5 which included the total number of 850 subjects.

### Data collection

During the first visit, subjects completed a questionnaire designed to assess the participants' demographics including age (year), gender, body weight, height, waist circumference,body mass index (BMI), physical activity (low active, moderate active, extremely active), educational level (illiterate, under diploma = uncompleted primary or secondary education, diploma = completed secondary education, educated = bachelor's degree or higher), marital status (single, divorced, dead spouse, married), job status (employed, retired, house-keeper, or unemployed), and smoking status (never smoked, former smoker, current smoker).

### Anthropometric measurements

Body weight was measured using a standard body weight scale (Seca 707; Seca GmbH & Co. KG., Hamburg, Germany, measurement accuracy ± 100 g). Participant’s height was measured using a wall stadiometer with a precision of 1 cm (Seca, Germany). The BMI was calculated as weight in kilograms divided by the square of height in meters. Waist circumference was measured with a tape measure to the nearest 0.1 cm between the iliac crest and the lowest rib during exhalation. Hip circumference was recorded at maximal point, over light clothing, using a non-stretch tape measure and without exerting any pressure on body surface. Obesity was defined as BMI ≥ 30 kg/m2 [24].

### Dietary assessment

Dietary intakes of participants were assessed by using three 24-h dietary recalls. Trained dietitians completed the first 24-h dietary recall by face-to-face interview at the first visit at each health center. The other two 24-h dietary recalls were completed at random days including one weekend, by telephone interviews. All 24-h dietary recall interviews were carried out by same trained dietitians. For dietary analysis, daily intakes of all food items obtained from 24-h dietary recalls were computed and then were converted into grams by using household measures [25]. The 3-day dietary intakes were summed and then averaged over the three days. Dietary intakes were expressed as food groups including total grains, fruit, vegetables, green-leafy vegetables, red/yellow vegetables, legumes, nuts, red meat, processed meat, poultry, fish, low- and high-fat dairy products, egg, soft drinks, salty snack, and solid and liquid oils. Liquid oil includes vegetable oils that were liquid in room temperature. Solid oil includes animal fat and hydrogenated vegetable oils that were solid in room temperature.

### Meals definition

Breakfast was defined as a meal eaten between 05:00 and 11:00 [26].

Lunch was predefined as a large meal eaten between 12:00 and 16:00 [26].

Dinner was defined as a large meal eaten between 17:00 and 23:00 [26].

### Physical activity assessment

Physical activity was assessed by using a validated short form of the International Physical Activity Questionnaire (IPAQ) [27]. Accordingly, IPAQ scores were categorized as ‘low physical activity’ (point score < 600 MET-min/week), ‘moderate physical activity’ (point score between 600 and 3000 MET-min/week) and ‘high physical activity’ (point score > 3000 MET-min/week) [28].

### DQI-I construction

Meal-based Diet quality was assessed based on the DQI-I that included four major dietary components [14]. The first component was variety, which included the overall variety of different food groups (meats and meat products, fish and shellfish, eggs, pulses and pulse products; milks and milk products; vegetables; fruits; grains) and the within-group variety of protein sources (meats and meat products, fishes and shellfishes, eggs, pulses and pulse products, milks and milk products), with a score ranging from 0 to 20 points. The second component was adequacy of intake (amounts of vegetables, fruits, grains, fiber, protein, Fe, Ca, and vitamin C), with a score ranging from 0 to 40 points. The third component was moderation (total fat, saturated fat, cholesterol, sodium, and empty calorie foods), with a score ranging from 0 to 30 points. The fourth component was overall balance (macronutrient ratio and fatty acid ratio), with a score ranging from 0 to 10 points. The total DQI-I score ranged from 0 to 100, with higher scores denoting better diet quality.

### Statistical analysis

Participants were categorized based on tertiles of DQI-I. Higher tertiles of DQI-I demonstrate higher diet quality compared to lower tertiles. The general characteristics and Dietary intakes of study subjects among tertiles of DQI-I were examined using analysis of variance (ANOVA) for continuous variables, and Chi-squared (χ^2^) for categorical variables. We used analysis of covariance (ANCOVA) to compare adjusted means of BMI across the DQI-I tertiles. The multiple linear regression analysis was used to evaluate the association between DQI-I with BMI based on sex in each meal after adjusting for possible confounders. Furthermore, odds ratio (OR) and 95% confidence interval (CI) of obesity were estimated through binary logistic regression analysis in two models. Model I adjusted for age, physical activity, socioeconomic status, and smoking. Model II was adjusted for confounders in Model I plus energy intake. All statistical analyses were performed using the Statistical Package for the Social Sciences (SPSS version 16; SPSS Inc.). P value less than 0.05 was considered statistically significant.

## Results

Table 1, shows anthropometric and demographic characteristics of the participants. The mean (± SD) of age, BMI, WC and WHR were 42.35(± 10.90) years, 27.32(± 5.61) kg/m2, 89.09 (± 12.04) cm and 0.86(± 0.11), respectively. The general characteristics of participants according to the tertiles of DQI for breakfast, lunch and dinner meals are presented in Table 2. In the dinner meal, participants in the top tertile of DQI compared to the bottom tertile, significantly had greater body weight (74.27 ± 14.54 vs 70.41 ± 12.54, *P* < 0.001). Furthermore, in both breakfast and dinner meal, men had greater adherence to DQI than women (*P* < 0.001). Also, the education status was different across the DQI tertiles for breakfast (*P* < 0.001).

**Table 1:** Characteristics of the investigating subjects

Variable	Mean	SD	Minimum	Maximum
Anthropometric/demographic variables				
Age (years)	42.35	10.90	18	67
Body weight (Kg)	72.09	13.88	37.00	147.00
Height (cm)	162.64	8.99	95.00	190.00
BMI (Kg/m^2^)	27.32	5.61	14.89	110.80
WC (cm)	89.09	12.04	31.00	138.00
HC (cm)	103.48	11.41	36.14	150.00
WHR	0.86	0.11	0.51	3.32

*SD* Standard deviation, *BMI* Body mass index, *WC* Waist Circumference, *HC* Hip Circumference, *WHR* Waist Hip Ratio

**Table 2:** General characteristics of study participants across the tertiles of DQI-I

Variables												
	DQI-I^*^ (Breakfast)				**DQI-I (Lunch)**				**DQI-I (Dinner)**			
	**T1 *N* = 281**	**T2 *N* = 282**	**T3 *N* = 283**	P^**^	**T1 *N* = 280**	**T2 *N* = 284**	**T3 *N* = 278**	***P***	**T1 *N* = 282**	**T2 *N* = 281**	**T3 *N* = 284**	***P***
**Age**	42.73 ± 10.16	42.15 ± 10.97	41.60 ± 10.58	0.20	42.43 ± 10.55	42.1 ± 10.66	41.92 ± 10.57	0.57	41.82 ± 10.95	42.42 ± 10.32	42.22 ± 10.53	0.65
**BMI**	27.38 ± 4.32	26.98 ± 4.82	27.23 ± 4.36	0.70	27.04 ± 4.45	27.44 ± 4.60	27.18 ± 4.41	0.69	26.90 ± 4.63	27.18 ± 4.29	27.56 ± 4.56	0.07
**Height**	1.62 ± 8.84	1.62 ± 8.95	1.63 ± 8.61	0.15^a^	1.62 ± 8.72	1.63 ± 8.85	1.62 ± 8.88	0.44	1.61 ± 8.45	1.62 ± 8.77	1.63 ± 9.14	0.06
**Weight**	72.75 ± 12.82	71.02 ± 13.93	73.09 ± 14.28	0.76	72.18 ± 14.19	73.10 ± 13.48	71.75 ± 13.46	0.71	70.41 ± 12.54	72.29 ± 13.68	74.27 ± 14.54	< 0.001
**Waist circumference**	89.62 ± 10.40	87.74 ± 12.13	89.79 ± 12.28	0.86	89.41 ± 12.09	88.81 ± 11.78	89.19 ± 11.02	0.81	87.92 ± 11.32	89.22 ± 11.63	90.17 ± 11.95	0.07
**Gender**				< 0.001				0.44				< 0.001
Male (%)	26(17.7)	49(33.3)	72(49.0)		42(32.3)	52(35.6)	52(35.6)		26(34.8)	49(29.3)	72(49.0)	
Female (%)	255(36.5)	233(33.3)	211(30.2)		238(34.2)	232(33.3)	226(32.5)		256(36.6)	232(33.1)	212(30.3)	
**Smoking**				0.07				0.16				
Not smoking (%)	263(33)	264(33.1)	271(34)		271(34.1)	266(33.5)	257(32.4)		264(33)	272(34)	263(32.9)	0.75
Quit smoking (%)	6(42.9)	8(57.1)	0(0.00)		2(14.3)	7(50.0)	5(35.7)		5(33.3)	4(26.7)	6(40)	
Smoking (%)	8(28.6)	8(28.6)	12(42.9)		5(17.9)	10(35.7)	13(46.4)		9(32.1)	7(25)	12(49.2)	
**Education**				< 0.001				0.78				0.47
Illiterate (%)	16(28.6)	19(33.9)	21(37.5)		19(33.9)	17(30.4)	20(35.7)		16(28.6)	17(30.4)	23(41.1)	
Under diploma (%)	61(31.1)	71(36.2)	64(32.3)		65(33.2)	59(30.1)	72(36.7)		64(32.3)	71(35.9)	63(31.8)	
Diploma (%)	98(33.7)	99(34.0)	94(32.3)		99(34.1)	100(34.5)	91(31.4)		88(30.1)	104(35.6)	100(34.2)	
Educated (%)	102(34.3)	91(30.6)	104(35.0)		95(32.3)	107(36.4)	92(31.3)		109(36.9)	89(30.2)	97(32.9)	
**Job status**				0.08				0.14				0.44
Employee (%)	104(33.4)	113(36.3)	94(30.2)		95(31.0)	110(35.9)	101(33.0)		100(32.6)	105(34.2)	102(33.2)	
Retired	19(40.4)	19(40.4)	9(19.1)		9(19.1)	20(42.6)	18(38.3)		21(44.7)	11(23.4)	15(31.9)	
Unemployed	10(22.2)	21(46.7)	14(31.1)		20(45.5)	10(22.7)	14(31.8)		11(24.4)	13(28.9)	21(46.7)	
Housekeeper (%)	174(32.8)	168(31.8)	188(35.5)		154(35.2)	142(32.4)	142(32.4)		145(32.9)	152(34.5)	144(32.7)	
**Marriage**				0.17				0.86				0.63
Single (%)	58(35.4)	56(34.1)	50(30.5)		39(34.8)	38(33.9)	35(31.2)		226(33.3)	227(33.5)	225(33.2)	
Divorced	3(25)	5(41.7)	4(33.3)		3(25)	3(25)	6(50)		6(50.0)	2(16.7)	4(33.3)	
Dead spouse	12(31.6)	14(36.8)	12(31.6)		15(39.5)	12(31.6)	11(28.9)		14(36.8)	11(28.9)	13(34.2)	
Married (%)	220(32.4)	226(33.3)	232(34.2)		221(32.8)	230(34.1)	223(33.1)		25(31.7)	56(34.1)	56(34.1)	
**Physical activity**				0.26				0.62				0.50
Low activity (%)	158(36.1)	145(33.1)	135(30.8)		142(32.3)	167(38.0)	131(29.8)		138(31.2)	161(36.3)	144(32.5)	
Moderate Active (%)	98(30.3)	105(32.5)	120(37.2)		104(32.5)	106(33.1)	110(34.4)		111(31.5)	100(30.3)	108(33.8)	
Extremely active (%)	21(27.6)	28(36.8)	27(35.5)		23(30.3)	27(35.5)	26(34.2)		27(35.5)	20(26.3)	29(38.2)	

^*^DQI-I diet quality index-international

^**^P value less than 0.05 was considered significant

^***^All values are mean ± SD or reported frequency (percentage)

^a^*P* values result from ANOVA for quantitative variables and χ2 test for qualitative variables

Table 3. details the mean and standard deviation of dietary intakes of participants across the tertiles of DQI scores for breakfast, lunch and dinner. Adherence to higher DQI for breakfast was significantly associated with higher intake of energy (*P* < 0.001), carbohydrate (*P* < 0.001), fat (*P* = 0.007), vitamin C (*P* < 0.001), calcium (*P* = 0.03) and iron (*P* < 0.001) and also associated with lower intake of SFA (*P* < 0.001) and MUFA(*P* = 0.04). Adherence to higher DQI for lunch was also associated with more intake of fat (*P* < 0.001), fiber (*P* < 0.001), vitamin C (*P* < 0.001), calcium (*P* < 0.001) and iron (*P* < 0.001) and correlated to lower consumption of cholesterol (*P* < 0.001), SFA (*P* < 0.001), PUFA (*P* < 0.001), MUFA (*P* < 0.001) and sodium (*P* < 0.001). Participants in the highest tertile of DQI for dinner consumed higher levels of energy (P < 0.001), carbohydrate (*P* < 0.001), fiber (*P* < 0.001), protein (*P* < 0.001), sodium (*P* = 0.01), vitamin C (*P* < 0.001), calcium (*P* < 0.001) and iron (*P* < 0.001) and they had a lower intake of, fat (*P* < 0.001), cholesterol (*P* < 0.001) SAFA (*P* < 0.001), PUFA (*P* < 0.001) and MUFA (*P* = 0.03).

**Table 3:** Dietary intakes of study participants across the tertiles of DQI-I

Variables	DQI-I (breakfast)				DQI-I (lunch)				DQI-I (dinner)			
	**T1 *N* = 281**	**T2 *N* = 282**	**T3 *N* = 283**	***P***^**c**^	**T1 *N* = 280**	**T2 *N* = 284**	**T3 *N* = 278**	***P***	**T1 *N* = 282**	**T2 *N* = 281**	**T3 *N* = 284**	***P***
**Energy**^**a**^	355.81 ± 127.68^b^	403.12 ± 127.31	497.23 ± 168.40	< 0.001	455.90 ± 171.22	544.98 ± 161.07	605.94 ± 170.91	0.30	1543.8 ± 358.39	1633.2 ± 343.55	1707 ± 426.74	< 0.001
**Carbohydrate**	59.47 ± 19.82	68.74 ± 25.96	85.00 ± 35.30	< 0.001	66.20 ± 35.19	66.21 ± 23.49	69.62 ± 44.73	0.55	57.77 ± 21.30	73.81 ± 31.24	86.47 ± 42.30	< 0.001
**Fat**	14.99 ± 22.63	12.54 ± 5.99	13.20 ± 9.73	0.007	22.39 ± 8.80	22.43 ± 9.15	22.49 ± 7.87	< 0.001	18.16 ± 9.20	18.37 ± 9.02	15.81 ± 7.79	< 0.001
**Fiber**	6.02 ± 2.99	9.69 ± 4.12	1.02 ± 2.99	0.52	5.97 ± 3.43	7.47 ± 3.90	8.27 ± 5.29	< 0.001	5.66 ± 2.70	7.59 ± 5.43	9.54 ± 7.67	< 0.001
**Protein**	11.24 ± 14.24	12.07 ± 4.98	16.91 ± 22.14	0.31	57.04 ± 17.33	56.67 ± 20.98	52.96 ± 28.26	0.12	15.22 ± 6.98	19.42 ± 8.48	21.68 ± 7.52	< 0.001
**Cholesterol**	65.51 ± 5.82	62.34 ± 5.93	57.18 ± 5.38	0.05	89.63 ± 7.08	69.15 ± 5.59	53.09 ± 5.20	< 0.001	89.25 ± 7.29	74.46 ± 64.70	60.80 ± 55.12	< 0.001
**SAFA**	6.25 ± 6.27	5.67 ± 2.84	5.27 ± 2.65	< 0.001	5.75 ± 2.91	5.17 ± 3.98	4.04 ± 265	< 0.001	4.98 ± 2.27	4.88 ± 3.02	4.03 ± 2.27	< 0.001
**PUFA**	2.55 ± 6.04	2.32 ± 1.72	2.50 ± 2.07	0.67	8.36 ± 3.28	7.65 ± 2.90	7.00 ± 3.74	< 0.001	7.27 ± 4.04	6.67 ± 4.59	5.95 ± 3.15	< 0.001
**MUFA**	18.45 ± 8.96	17.57 ± 12.64	18.45 ± 8.96	0.04	7.83 ± 3.89	7.71 ± 1.05	5.93 ± 5.2`	< 0.001	8.63 ± 2.99	5.94 ± 3.41	4.91 ± 3.07	0.03
**Sodium**	567.70 ± 251	426.38 ± 214.95	658.98 ± 428.17	0.55	892.29 ± 41.94	766.98 ± 38.48	729..67 ± 36.95	< 0.001	799.15 ± 46.35	883.14 ± 70.56	810.36 ± 43.66	0.01
**Vit C**	16.05 ± 1.39	17.30 ± 1.68	26.48 ± 2.88	< 0.001	21.41 ± 1.56	27.87 ± 2.33	33.50 ± 3.01	< 0.001	20.51 ± 1.67	30.61 ± 2.74	39.56 ± 2.87	< 0.001
**calcium**	175.39 ± 111.7	188.98 ± 83.34	216.17 ± 114.35	0.03	168.12 ± 104.49	187.94 ± 115.16	204.36 ± 129.75	< 0.001	15.73 ± 1.02	21.95 ± 1.32	25.17 ± 1.46	< 0.001
**Iron**	2.26 ± 0.89	2.84 ± 1.37	4.40 ± 1.03	< 0.001	5.03 ± 2.27	6.20 ± 1.20	6.87 ± 5.25	0.06	3.67 ± 2.29	5.54 ± 6.81	7.60 ± 6.65	< 0.001

*DQI-I* diet quality index-international, *SFA* saturated fatty acid, *PUFA* polyunsaturated fatty acid, *MUFA* monounsaturated fatty acid, *SD* standard deviation

^a^Energy was not adjusted

^b^All values are mean ± SD

^c^*P* values obtained from ANCOVA

The results of ANCOVA analysis shows the BMI differences across DQI tertiles in both male and female populations separately in Table 4. The results presented in crude and two adjusted models; the model 1 was adjusted for age, physical activity, socioeconomic status and smoking and model 2 was adjusted for age, physical activity, socioeconomic status and smoking plus energy intake. In the breakfast meal, a non-significant difference was observed in BMI across the tertiles of DQI in adjusted model 2 for both women (*P* = 0.90) and men (*P* = 0.83). Moreover, in the lunch meal, no significant difference was found regarding BMI across DQI categories in model 2 and both women (*P* = 0.54) and men (*P* = 0.66). In the dinner meal, we also found no significant differences in mean of BMI across tertiles of DQI in model 2 and both women (*P* = 0.22) and men (*P* = 0.81) population.

**Table 4:** Means of BMI across the tertiles of DQI-I based on sex in each meal

Variables	DQI-I^**a**^ (Breakfast)				DQI-I (Lunch)				DQI-I (Dinner)			
	T1 N:254	T2 N:231	T3 N:211	*P*	T1 N:237	T2 N:231	T3 N:225	*P*	T1 N:253	T2 N:232	T3 N:212	*P*
Women												
Crude	27.38 ± 4.20^e^	27.06 ± 4.84	27.12 ± 4.14	0.51	27.01 ± 4.35	27.40 ± 4.47	27.23 ± 4.35	0.62	26.94 ± 4.47	27.01 ± 4.17	27.77 ± 4.52	0.09
^** b**^ Model 1	27.38 ± 4.23	27.10 ± 4.84	27.09 ± 4.15	0.79	27.01 ± 4.37	27.47 ± 4.46	27.22 ± 4.37	0.54	26.88 ± 4.56	27.20 ± 4.20	27.61 ± 4.46	0.19
^** c**^ Model 2	27.38 ± 4.23	27.10 ± 4.84	27.09 ± 4.15	0.90	27.01 ± 4.37	27.46 ± 4.47	27.22 ± 4.37	0.54	26.88 ± 4.56	27.20 ± 4.20	27.61 ± 4.46	0.22
Men	N:27	N:51	N:72		N:43	N:53	N:53		N:29	N:49	N:72	
Crude	27.39 ± 5.47	26.58 ± 4.75	27.58 ± 4.94	0.87	27.20 ± 5.01	27.60 ± 5.17	26.98 ± 4.70	0.81	26.92 ± 5.14	27.17 ± 4.77	27.55 ± 5.05	0.53
Model 1	27.39 ± 5.47	26.58 ± 4.75	27.38 ± 4.67	0.74	27.20 ± 5.01	27.60 ± 5.17	26.98 ± 4.70	0.78	26.92 ± 5.14	27.30 ± 4.73	27.73 ± 5.08	0.89
Model 2	27.39 ± 5.47	26.58 ± 4.75	27.38 ± 4.67	0.83	27.20 ± 5.01	27.60 ± 5.17	26.98 ± 4.70	0.66	25.75 ± 3.50	27.61 ± 5.78	27.47 ± 4.79	0.81

^a^*DQI-I* diet quality index-international

^b^Model 1: adjusted for age, educational level, occupation, marriage, smoking status, physical activity and other meals (breakfast or lunch or dinner, where appropriate)

^c^Model 2: adjusted for age, educational level, occupation, marriage, smoking status, physical activity, other meals (breakfast or lunch or dinner, where appropriate) and energy intake

^d^Data are presented as mean ± standard deviation. P Obtained from ANCOVA test

Results of multiple linear regression for the association between DQI-I and BMI based on sex in each meal are reported showed in Table 5. Results were adjusted for age, educational level, occupation, marriage, smoking status, physical activity and energy intake. The results of multiple linear regression did not show any significant association between DQI-I and BMI in both men and women populations. Gender-based analysis also showed that among women, a non-significant relationship was showed between DQI and BMI in breakfast (R2 = 0.03, P = 0.23), lunch (R2 = 0.03, *P* = 0.42) and dinner meal (R2 = 0.03, *P* = 0.15). A non-significant association was also found between DQI and BMI in breakfast (R2 = 0.07, *P* = 0.70), lunch (R2 = 0.07, *P* = 0.61) and dinner meal (R2 = 0.06, *P* = 0.30) in men.

**Table 5:** Multiple linear regression analysis of the associations between DQI-I and BMI based on sex in each meal

BMI				
	**β ± SE**	**95% CI**	**Partial R**^**2**^	***P* Value**
**Women**				
R^2^ model = 0.03				
** DQI-I**^**a**^**(breakfast)**	-0.04 ± 0.02	-0.06–0.01	0.03	0.23
** DQI-I (lunch)**	0.01 ± 0.02	-0.03–0.04	0.03	0.42
** DQI-I (dinner)**	0.03 ± 0.02	-0.02–0.05	0.03	0.15
**Men**				
R^2^ model = 0.06				
** DQI-I (breakfast)**	0.005 ± 0.05	-0.10–0.10	0.07	0.70
** DQI-I (lunch)**	-0.01 ± 0.04	-0.09–0.08	0.07	0.61
** DQI-I (dinner)**	0.03 ± 0.04	-0.06–0.10	0.06	0.30

^a^*DQI-I* diet quality index-international, *β* standardized coefficients, *SE* standard error, *CI* confidence interval, *R2* R square results are adjusted for age, educational level, occupation, marriage, smoking status, physical activity and energy intake

Odds ratio (OR) and 95% confidence intervals (CIs) for obesity across the tertiles of DQI are indicated in Table 6. The odds of obesity across tertiles of DQI was not statistically different for any of meals, even separately by sex.

**Table 6:** Odds ratio (95% CI) for obesity according to tertiles (T) of DQI-I

Variables	DQI-I^a^ (breakfast)				DQI-I (Lunch)				DQI-I (Dinner)			
	**T1**	**T2**	**T3**	**p-trend**	**T1**	**T2**	**T3**	**p-trend**	**T1**	**T2**	**T3**	**p-trend**
**Women**	**N:254**	**N:231**	**N:211**		**N:237**	**N:231**	**N:225**		**N:253**	**N:232**	**N:212**	
**Obesity**^**b**^												
** Crude**	1	1.27(0.78–2.05)	1.10(0.71–1.70)	0.25	1	0.98(0.63–1.50)	1.18(0.78–1.78)	0.72	1	0.78(0.52–1.18)	0.77(0.50–1.17)	0.25
** Model I**^**c**^	1	1.30(0.84–2.02)	1.12(0.71–1.74)	0.22	1	0.90(0.58–1.40)	1.29(0.83–1.99)	0.61	1	0.78(0.51–1.20)	0.76(0.49–1.17)	0.29
** Model II**^**d**^	1	1.28(0.83–1.99)	1.11 (0.71–1.73)	0.20	1	0.91(0.58–1.41)	1.29(0.84–1.99)	0.65	1	0.78(0.41–1.20)	0.76(0.49–1.17)	0.22
** Men**	**N:27**	**N:51**	**N:72**		**N:43**	**N:53**	**N:53**		**N:29**	**N:49**	**N:72**	
** Crude**	1	1.52(0.56–4.16)	0.63(0.24–1.60)	0.66	1	1.62(0.59–4.42)	1.75(0.59–4.60)	0.45	1	0.62(0.18–2.07)	1.24(0.53–2.88)	0.63
** Model I**	1	1.30(0.47–3.84)	0.91(0.34–2.44)	0.71	1	1.33(0.47–3.73)	1.24(0.45–3.32)	0.58	1	0.61(0.18–2.12)	1.22(0.51–2.91)	0.86
** Model II**	1	1.18(0.39–3.58)	0.85(0.31–2.34)	0.84	1	1.47(0.51–4.19)	1.26(0.46–3.47)	0.46	1	0.72(0.20–2.60)	1.33(0.53–3.25)	0.82

^a^ *DQI-I* diet quality index-international

^b^ Obesity is defined as body mass index (BMI) ≥ 30 kg/m2

^c^ Model 1: adjusted for age, educational level, occupation, marriage, smoking status, physical activity and other meals (breakfast or lunch or dinner, where appropriate)

^d^ Model 2: adjusted for age, educational level, occupation, marriage, smoking status, physical activity, other meals (breakfast or lunch or dinner, where appropriate) and energy intake

*P* value: less than 0.05 was considered significant

^e^ Results are presented as odds ratios (OR) and 95% confidence intervals (CI)

## Discussion

This is the first study that used a meal specified to assess the potential association of the DQI, representing patterns of lifestyle and dietary intake, in a sample of Iranian adults, and obesity in adults. The main analysis of dietary intakes across the tertiles of DQI scores for each meal provided evidence that the overall diet quality of Iranian adults in this study has a relatively healthy pattern with lower consumption of cholesterol and SFA along with higher intake of micronutrients like vitamin C, calcium and iron. In the all three meals we found no significant differences for BMI across the DQI groups in adjusted models for both women and men. The chance of having obesity also was not different across DQI tertiles.

Understanding the nutritional composition of meals and the ways in which different meal patterns make an impact on diet quality might help to elucidate important diet–disease relationships. The relationship between dietary patterns in adults with nutrient intake and diet quality is essential to determine if they represent the health and diversity of the entire diet. As a holistic view, these behaviors include several components that work synergistically on health and disease [29, 30]. In this regard, in recent years, dietary quality indices have been developed to assess the requirement of nutritional research [31].

In various countries including France [32], USA [33–35], Iran [16], Guatemala [36] and Spain [37], the DQI was evaluated. In agreement with our findings over a 10-year follow-up period among Spanish men and women Funtikova et al. [37] reported a ten-point increment in the DQI predicted a 3·2 cm reduction in WC after adjustment for confounders, but there was no significant prediction of BMI. Meanwhile in the Tehran Lipid and Glucose Study for both longitudinal and cross-sectional analyses, no significant association were observed [16]. In contrast, Quatromoni et al. [34] found an inverse, linear relationship between better adherence to DQI and lower weight gain over an 8-year follow up in the Framingham Offspring Study. Also, after 13 years of follow up in French Lassale et al. [32] study observed an inverse prediction in men with no prediction in women. In addition, in a cross-sectional study from Guatemala, DQI-I was positively correlated with BMI and WC in both men and women [36]. Thus, according to literature review, studies assessing DQI or its modified versions revealed controversial and sex-specific findings.

Conflicting findings on the association between general obesity and diet quality indices can be explained by several reasons. Most of these dietary scores, which assessed adherence to dietary guidelines (e.g., HEI, DQI, and DGAI), were designed for the U.S. population. However, the population may not be able to assess the overall quality of the diet until more information is known about the consumption patterns. In addition, there are differences in the scoring models of indices, based on dietary guidelines. Simultaneously, measures of diet quality in developing countries are more complex to interpret; it is also complex in such countries to assess diet quality in terms of both micronutrient adequacy and prevention of overweight, indicating the need for better measures of diet quality specifically for these populations. The major issues in developing countries are both under- and over-nutrition [38]. Furthermore, the range of the higher categories was relatively limited compared to the lower categories, indicating that people in the upper and lower groups did not clearly show differences in dietary patterns and could attenuate the association between diet and obesity. Conflicting results may also be due to the fact that overweight individuals adopt a healthier diet to manage their weight, and the effect of a healthy diet as assessed by scores on their obesity status hence could not be detected. In addition, the population that the index is developed for is important. Hence, it is clear that specific indices can be evaluated only in specific populations [38].

We did not find gender-specific associations between DQI and BMI or obesity. However, gender differences in diet quality findings in other studies has been reported [34] that may be explained by some factors. Women tend to gain more weight over time than men do. This observation reflects the important influence of other contributors to energy balance and weight management that are gender-specific, including menopausal status [34]. Menopausal women tend to gain more weight over time than men, resulting from the potentially confounding effect of hormonal changes.

The present study has important strengths. To the best of the authors’ knowledge, this is the first study to assess the relationship between DQI-I at meals and obesity in Iranian population. In this study, we had a sufficient sample size. Also, this sampling was done from different parts of health centers in Tehran. Then, results can be generalizable to all the Tehranian population and also as Tehran is the Capital of Iran and has a multiethnic population, and population of Tehran are considered as a representative of Iran. However, some limitations of the present study must be addressed. First, the cross-sectional design of the study also needs to be considered when interpreting the findings. Second, the use of 24-h dietary recall data may have been subjected to recall bias of self-reported measurement error due to within-subject variations. In addition, 24-h diet recall data is limited in its ability to capture dietary diversity. Finally, although we included a rich set of confounding variables in the models, some residual confounding may exist.

## Conclusion

In the present study, we failed to find any significant association between meal-specified DQI with obesity. Diet quality may be an integral component of a broader obesity intervention strategy. Although better diet quality may not lead to decreased BMI for all individuals, it is important for health promotion; other researchers have demonstrated substantial health benefits from consuming high-quality diets. To examine the exact relationships, more research is needed to better design and evaluate diet quality indices for each population.

## Data Availability

The data that support the findings of this study are available from [Sakineh Shab-Bidar] but restrictions apply to the availability of these data, which were used under license for the current study, and so are not publicly available. Data are however available from the authors upon reasonable request and with permission of [Sakineh Shab-Bidar].

## Abbreviations

DQI-I: Diet quality index-international
BMI: Body mass index
WC: Waist circumference
WHR: Waist to hip ratio
IPAQ: International Physical Activity Questionnaire
